# CULTURAL ADAPTATION OF THE ADULT DAY SERVICE PLUS PROGRAM FOR HISPANIC/LATINO DEMENTIA CAREGIVERS

**DOI:** 10.1093/geroni/igac059.817

**Published:** 2022-12-20

**Authors:** Lauren Parker, Katherine Marx, Manka Knimbeng, Elma Johnson, Sokha Koeuth, Joseph Gaugler, Laura Gitlin

**Affiliations:** Johns Hopkins Bloomberg School of Public Health, Baltimore, Maryland, United States; Johns Hopkins School of Nursing, Baltimore, Maryland, United States; University of Minnesota, Minneapolis, Minnesota, United States; University of Minnesota School of Public Health, Minneapolis, Minnesota, United States; Drexel University, Philadelphia, Pennsylvania, United States; University of Minnesota, Minneapolis, Minnesota, United States; Drexel University, Philadelphia, Pennsylvania, United States

## Abstract

Although Hispanic/Latinos are at disproportionate and increased risk for Alzheimer’s disease and related dementias, few evidence-based supportive care interventions have been specifically developed for or adapted for this population. Adapting a supportive care intervention requires more than Spanish language translation and necessitates an understanding of cultural nuances and care preferences of Hispanic/Latino families and staff who implement the intervention. This paper reports on the cultural adaptation of the Adult Day Service Plus (ADS Plus) intervention for delivery by staff to Hispanic/Latino caregivers which was guided by the Cultural Adaptation Process Model. Also, using the Framework for Reporting Adaptations and Modifications-Enhanced (FRAME),we discuss: 1) when modifications were made, 2) who determined modifications needed, 3) what aspects of the intervention were modified, 4) the relationship to fidelity and how fidelity was maintained, and 5) reasons for modifications. Modifications to the delivery and content were changed to reflect values and norms of both the Hispanic/Latino staff and the caregivers they serve. As supportive interventions for dementia caregivers are developed and implemented into real world settings, inclusion of cultural elements may enhance research participation from Hispanic/Latino provider sites and caregivers. We suggest in this paper that cultural adaptation is an essential consideration in developing an intervention as well as adapting evidence-based previously tested interventions, and in implementation science. Cultural adaptation offers an important lens by which to identify contextual factors impacting intervention adoption interventions and needed adaptations to assure equity in the reach of evidence-based programs.

